# A Novel Panel of 80 RNA Biomarkers with Differential Expression in Multiple Human Solid Tumors against Healthy Blood Samples

**DOI:** 10.3390/ijms20194894

**Published:** 2019-10-02

**Authors:** Lucas Delmonico, John C. Obenauer, Fatir Qureshi, Gilda Alves, Mauricio Augusto Silva Magalhães Costa, Katherine J. Martin, Marcia V. Fournier

**Affiliations:** 1 Carlos Chagas Filho Institute of Biophysics, Federal University of Rio de Janeiro (UFRJ), Rio de Janeiro 21941-902, Brazil; 2 Rancho BioSciences, San Diego, CA 92127, USA; john.obenauer@ranchobiosciences.com; 3 Center for Biotechnology and Interdisciplinary Studies, Rensselaer Polytechnic Institute, Troy, NY 12180, USA; quresf2@rpi.edu; 4 Circulating Biomarkers Laboratory, Faculty of Medical Sciences, Rio de Janeiro State University, Rio de Janeiro 20550-170, Brazil; galvesbrown@gmail.com; 5 Americas Medical City, Barra da Tijuca, Rio de Janeiro 22775-001, Brazil; mamcosta@yahoo.com; 6 40 Beaver Pond Rd, Lincoln, MA 01773, USA; kjmartin620@gmail.com; 7 101 Riggs Avenue, West Hartford, CO 06107, USA

**Keywords:** genomics, gene profiling, gene expression, biomarkers, circulating tumor cells, breast cancer, RNA signature

## Abstract

The aim of this study was to identify genes with higher expression in solid tumor cells by comparing human tumor biopsies with healthy blood samples using both in silico statistical analysis and experimental validations. This approach resulted in a novel panel of 80 RNA biomarkers with high discrimination power to detect circulating tumor cells in blood samples. To identify the 80 RNA biomarkers, Affymetrix HG-U133 plus 2.0 microarrays datasets were used to compare breast tumor tissue biopsies and breast cancer cell lines with blood samples from patients with conditions other than cancer. A total of 859 samples were analyzed at the discovery stage, consisting of 417 mammary tumors, 41 breast lines, and 401 control samples. To confirm this discovery, external datasets of eight types of tumors were used, and experimental validation studies (NanoString n-counter gene expression assay) were performed, totaling 5028 samples analyzed. In these analyses, the 80 biomarkers showed higher expression in all solid tumors analyzed relative to healthy blood samples. Experimental validation studies using NanoString assay confirmed the results were not dependent of the gene expression platform. A panel of 80 RNA biomarkers was described here, with the potential to detect solid tumor cells present in the blood of multiple tumor types.

## 1. Introduction

Cancer mortality continues to be a major healthcare problem, despite of decades of advances in the area of oncology [1]. The reason for this problem is that before many cancers are discovered, they have already progressed and become drug-resistant or have metastasized [2]. However, the recent introduction of liquid biopsies using biomarkers found in blood has offered a valuable opportunity for development of clinical oncology tests to provide better diagnosis, surveillance, and prediction of outcomes, thereby reducing cancer mortality [3]. Blood sampling has advantages over solid biopsy, as it is widely accepted, readily repeated, convenient, minimally invasive, and of low cost. Furthermore, liquid biopsy could speed up the diagnostic process and potentially reduce health care costs by providing a reliable screening tool. Biomarkers in blood have the potential to detect a wide variety of primary tumors and metastases located throughout the body. These biomarkers include a variety of components, including circulating tumor cells (CTCs), molecules such as cell-free and tumoral DNA, microRNAs (miRNAs), and small organelles such as exosomes [4]. Although a number of technologies have been developed for the isolation and detection of these components, only a few of these have been successfully translated to the clinic because of shortcomings in sensitivity and specificity [4,5,6].

Tumor cell shedding begins early in tumor progression, once angiogenesis links the tumor and blood [7]. A number of studies have shown a detection of a range of 5 to 1281 CTCs per mL of whole blood in individuals with cancer [8], and these have often been cited as promising indicators of prognosis and treatment response [9,10]. About 5 CTCs per 7.5 mL blood are associated with worse outcome, and an increase with time is a strong independent predictor of rapid disease progression [10]. Furthermore, 10%–30% of patients with non-metastatic breast cancer have CTCs potentially capable of invasion and establishment of a secondary tumor site [11,12,13,14].

The isolation of CTCs is challenging, as it is necessary to enrich low levels of CTCs against a background of 10 million leukocytes and 5 billion erythrocytes per mL of blood [15]. The background also includes non-tumor epithelial cells, which can be found in blood. An additional problem is the intrinsic genetic heterogeneity of tumors across patient populations [15,16]. For example, well-established markers, such as estrogen receptor (ER) and Human Epidermal Growth Factor Receptor-type 2 (HER2/neu), are used to differentiate breast cancer types, but the expression of these receptors can change between primary tumor and CTCs [17,18]. In relation to the other components, circulating tumoral DNA (ctDNA), circulating free DNA (cfDNA), and exosomes, there is a greater range of tests on the market (Cobas Epidermal Growth Factor Receptor [EGFR] Mutation Test v2 from Roche Molecular Systems, Inc., CA, USA; CronixBiomedical Products from Chronix Biomedical, CA, USA; UltraSEEK colon and lung panels from Agena Bioscience, Inc., CA, USA; and ExoDx lung and prostate tests from Exosome Diagnostics, MA, USA), all of them focused on the detection of point mutations directly involved in the targeted therapy. Furthermore, despite the development of these tests, ctDNA represents a small proportion of the whole cfDNA isolated—0.1% to over 10% depending on tumor factors—which include size, stage, and tumor infiltration [4,19].

In this way, improvements in the sensitivity, accuracy, and reproducibility of current CTC detection tests are necessary for broader applications, as most of the techniques to count CTCs rely on a combination of isolation and detection steps. Isolation is generally accomplished by antibodies directed toward a single carcinoma-associated antigen, typically epithelial cell adhesion molecule (EpCAM). Following isolation, CTCs are distinguished by immunostaining for cytokeratins (e.g., cytokeratin 8 (CK8), cytokeratin 18 (CK18), cytokeratin 19 (CK19)). An example is the Food and Drug Administration (FDA)-approved CellSearch system (Menarini Silicon Biosystems). CTCs that do not express *EpCAM* are not detected by the CellSearch assay. Furthermore, the *EpCAM* expression rate is quite variable and can reach up to 50% negativity in mammary tumors, being more expressed in advanced tumors [20]. Also, it has been reported that *EpCAM* can be expressed in leukocytes adding a confounder factor [21]. There are other methods of CTCs isolation, but they depend on the phenotypic characterization of cells, which include density, size, and epithelial labeling [22,23].

The approach of comparing blood samples from cancer patients with health donors has been extensively used by other studies to identify circulating tumor cell markers. However, poor signal to noise ratio limited the application of such biomarkers [24,25]. In addition, many of the biomarkers identified with standard approaches were general epithelial markers such as EpCAM and KRT19. With the objective to expand the menu of blood biomarkers, we decided to undertake a new approach by directly comparing tumor samples with healthy blood to identify highly expressed genes that could (1) provide higher signal to noise ratio, and (2) provide tumor markers in addition to the well-known general epithelial markers. Then, we describe here a novel panel of 80 biomarkers to fill an unmet need for discrimination of tumor cells in blood. A total of 5028 samples including 8 cancer types were analyzed using in silico Affymetrix data analysis, and experimental validation was done using a custom-designed NanoString n-counter assay, confirming that the results are platform agnostic. These markers, rather than general epithelial markers, represent tumor gene expression profiles dominant in tumor cells in comparison to blood cells. The gene panel described here is innovative because it brings a combination of new and known biomarkers for detecting CTCs. The biomarkers described extend the perspective in the field of liquid biopsy, as they can be translated, combined, and adapted to enable other technologies.

## 2. Results

### 2.1. Discovery Set

**Figure 1:**
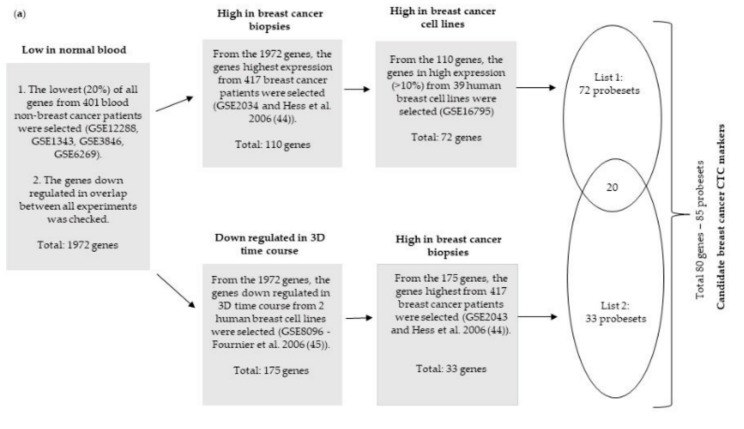

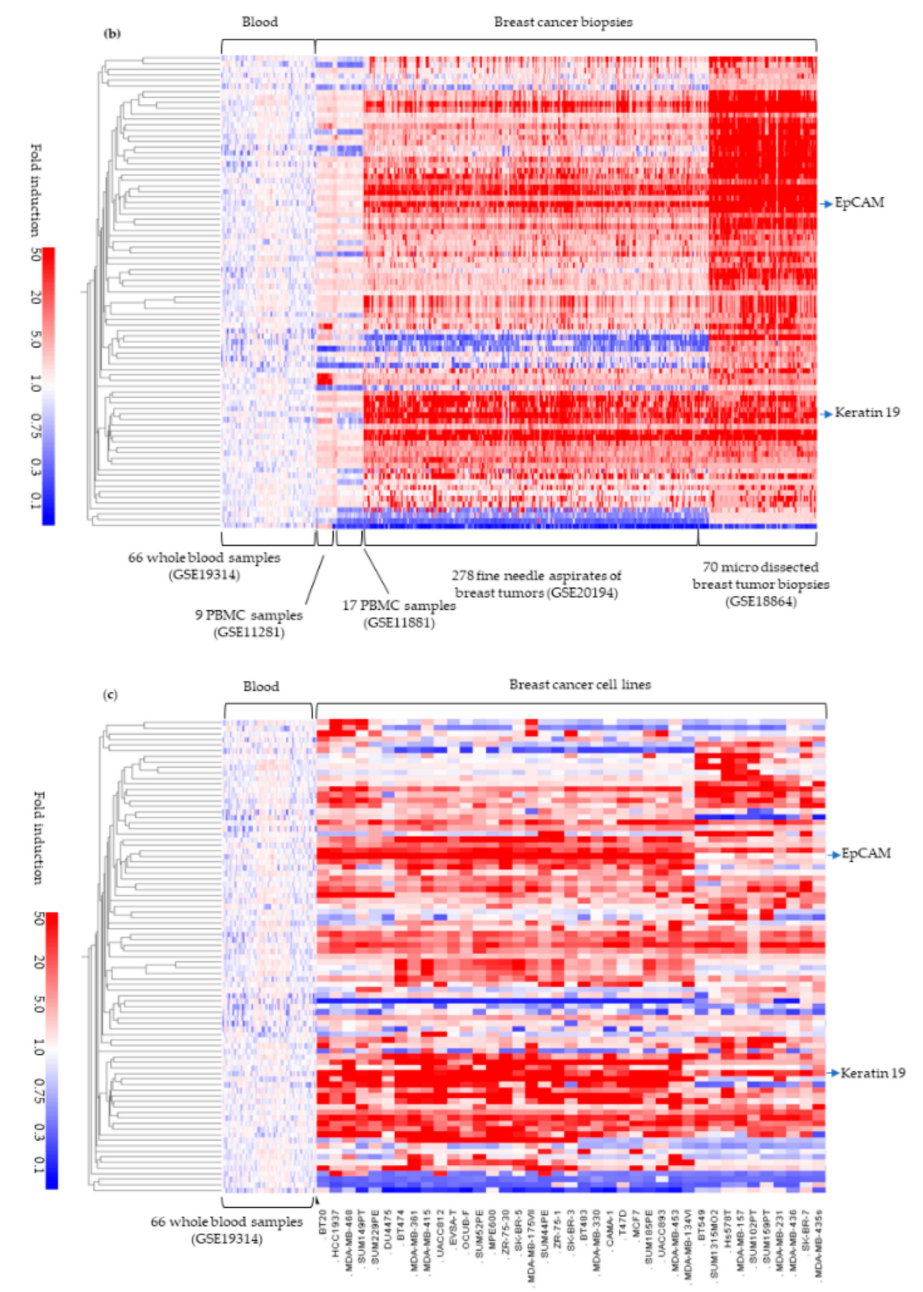
Scheme for the discovery of 80 genes and evaluation of expression in different datasets. (**a**) Scheme of selection for the discovery of the 80 genes. Three-dimensional (3D) culture assays allow phenotypic discrimination between nonmalignant and malignant mammary cells. Nonmalignant cells form polarized and acinus colonies attached to growth, while malignant cells form disorganized, proliferative and nonpolar colonies. (**b**) Heatmap showing the expression profile of the 80 genes in 348 breast cancer biopsies when normalized to non-breast cancer blood samples, including two datasets from peripheral blood mononuclear cells. (**c**) Heatmap showing the expression levels of the 80 genes in 39 breast cell lines when normalized to non-cancer blood samples. PBMC: peripheral blood mononuclear cell, CTC: circulating tumor cell.

The challenge in finding specific biomarkers for detecting CTCs in the bloodstream is in the ability to eliminate signs of gene expression from blood cells, such as leukocytes and erythrocytes, in addition to non-tumoral epithelial cells. To address this challenge, we analyzed multiple studies using genome-wide gene expression microarrays (Affymetrix HG-U133A) of breast tumor cells and compared them with blood samples from individuals with conditions other than cancer to find high expressing genes in tumor samples that are expressed in blood at the background level. A total of 859 samples were used in this comparison, including breast cancer tissue biopsy samples, breast cell lines, and control blood samples (Figure 1a) (see Methods section for the datasets analyzed). From this procedure, 85 Affymetrix probesets representing 80 genes were selected, all having negligible expression levels (expressed at the gene chip background level) in control blood samples and high levels in breast tumors. The high levels ranged from 10- to 300-fold greater than the controls (average biopsy/average normal blood). The list of genes, average expression, and fold changes of the aggregated specimens can be seen in Supplementary Materials Table S1.

### 2.2. External Validation in Breast Tumor Samples

The expression levels of the 80 genes were studied in two independent sets of breast tumor samples (samples containing 80% to 100% tumor content) and normalized to the median of an independent set of control samples. These two datasets were not used for the original selection procedure (see Methods Section). The total of 348 breast tumor samples were normalized to blood samples obtained from 66 patients with conditions other than cancer. Results showed that the great majority of the 80 genes were highly overexpressed in the tumors relative to the blood samples (Figure 1b). To confirm the specificity of expression of the 80 genes in breast tumors, we expanded the comparison of expression to include two peripheral blood mononuclear cell (PBMC) data sets of individuals with non-cancerous conditions. The normalized data demonstrate much higher expression of the 80 genes in breast tumors than in either whole blood or PBMCs (Figure 1b).

Of the 85 probesets (89%), 76 were expressed at least 20-fold greater in at least one breast tumor biopsy in comparison to blood controls. Although *EpCAM* is considered the gold standard biomarker for CTCs detection, we observed that levels of *EpCAM* were lower (within 20-fold of the microarray background or blood controls) in 10 of 70 (14%) of micro-dissected breast cancer biopsies; consequently, the use of this unique biomarker could result in false negative cases [26]. On the other hand, another important and known biomarker for detecting CTCs, *KRT19*, demonstrated more reliable detection of tumors evaluated here (Figure 1b). These results confirmed that the 80 genes selected are putative biomarkers for CTC.

### 2.3. External Validation in 39 Breast Cell Lines 

To further confirm the results, the expression levels of the 80 genes in 39 breast cancer cell lines when normalized to non-cancer blood samples showed that 73 of 85 probesets (86%) were overexpressed at least 20-fold in at least one cell line (Figure 1c). The levels of *EpCAM* were observed to be low in 10 of 39 (26%) of breast cell lines, which is consistent with results showing *EpCAM* alone does not offer a broad detection of tumor cells.

### 2.4. Experimental Validation in Breast Tissue Samples and Blood 

**Figure 2:**
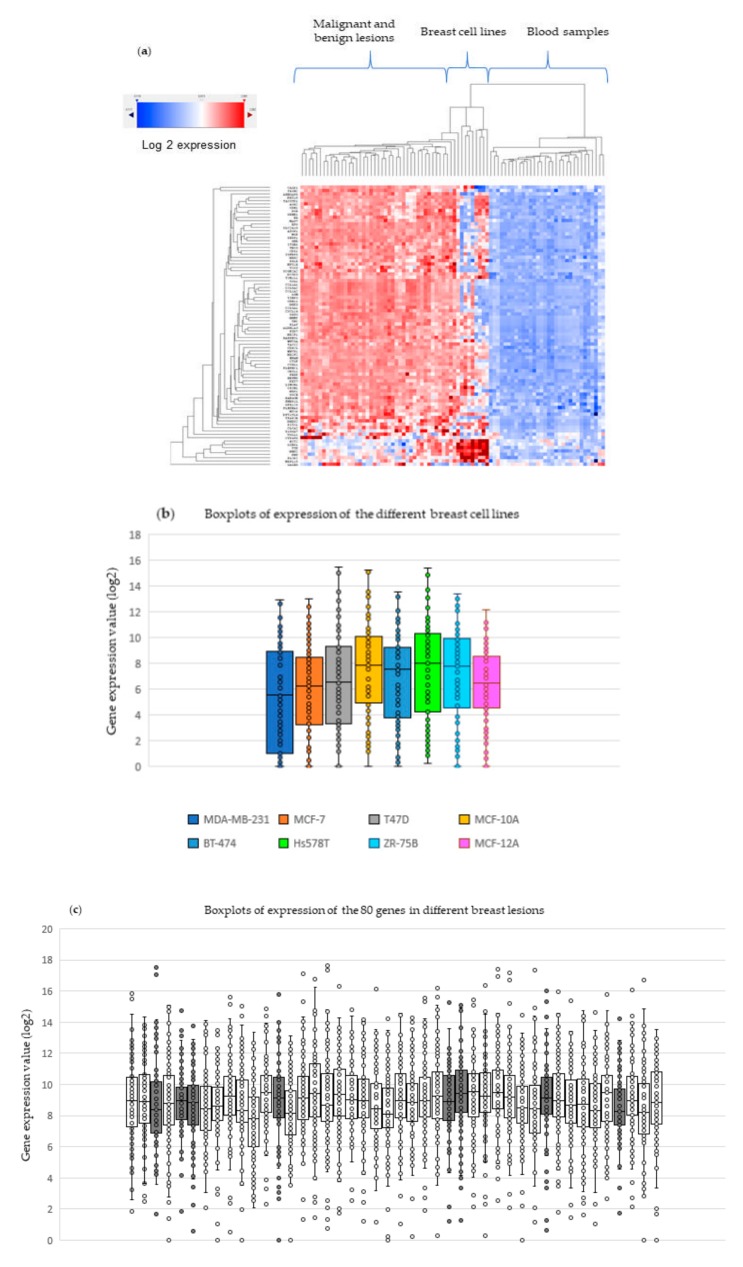

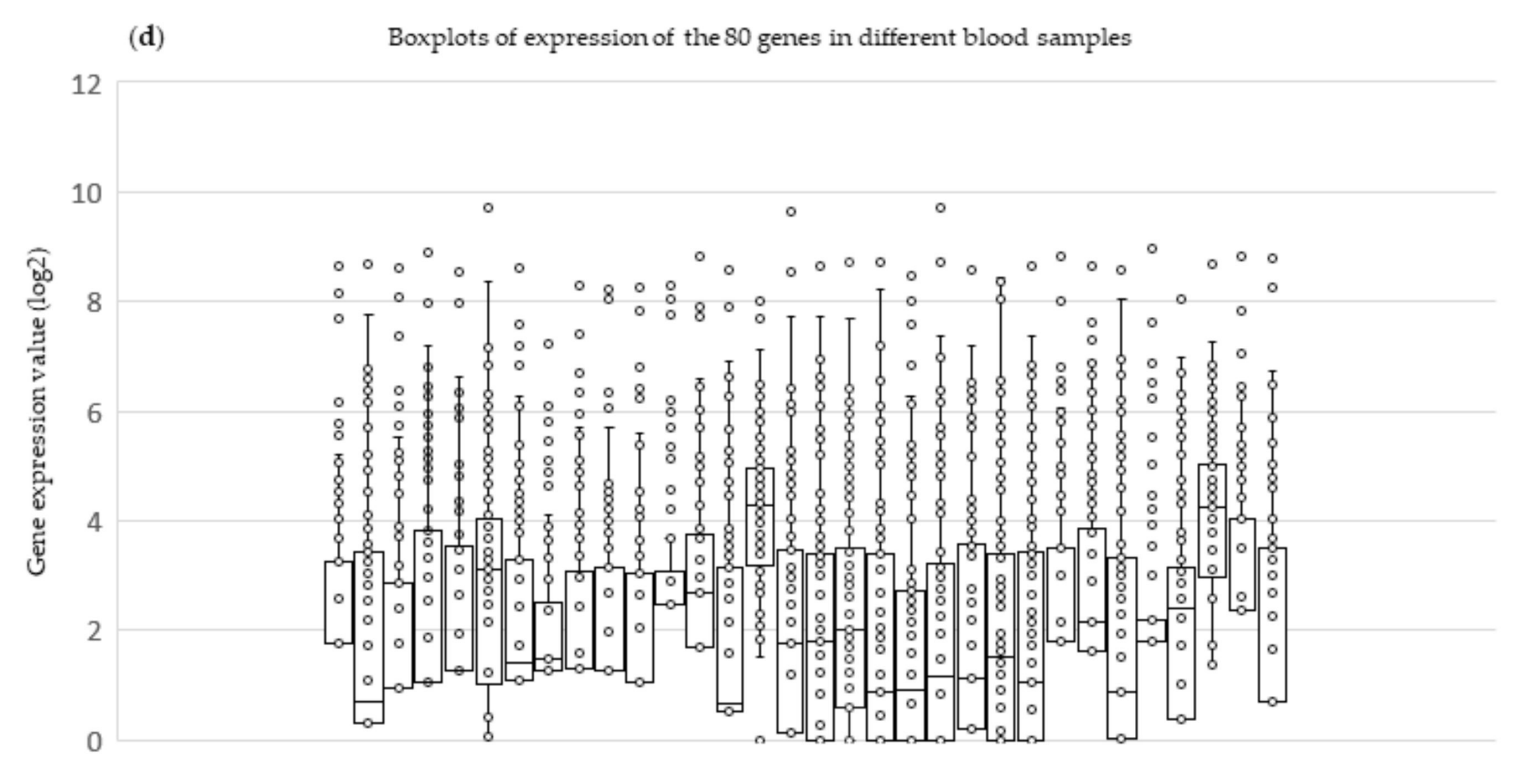
Experimental validation in breast cell lines, breast tissue, and blood samples. (**a**) Heatmap showing the expression levels of the 80 genes in breast lesions samples compared to the expression levels of breast cell lines and blood from patients with conditions other than cancer. (**b**) Boxplots showing the expression levels for each cell line available. (**c**) Boxplots showing the expression levels for each breast lesion (benign and malignant). Boxplots show the expression of the 80 genes in 44 breast tumors, including benign (*n* = 8) (gray boxes) and malignant lesions (*n* = 36) (white boxes). The means of expression (log2) are visible between 8 and 10. (**d**) Boxplots showing the expression levels for each blood sample (buffy coat). The boxplots show the expression of the 80 genes in 32 buffy coats from cases with conditions other than cancer. The expression means (log2), for the most part, are visible below 4.

To provide experimental validation of the novel 80-gene panel expression profiles, we performed a series of experiments using a custom-designed NanoString nCounter assay (see Methods Section). First, the levels of expression of the genes were evaluated in 44 samples of breast tissue biopsies (benign *n* = 8 and malignant *n* = 36) and second, in 8 different types of breast cancer cells lines selected from prior analysis. These expression data were normalized and compared to the levels of expression of 32 non-cancer blood samples sequentially obtained at UConn Health. Supplementary Materials Tables S2 and S3 show the demographic and histological data of the samples analyzed. Figure 2 shows the expression comparison of these samples as a heatmap (Figure 2a) and as boxplots (Figure 2b–d). It is possible to note the magnitude of expression of the 80 genes in the malignant and benign biopsies in relation to the blood. While tumors ranged between 8 to 10 in the log2 scale, and cell lines ranged from 6 to 9, the blood range was below 4 (background). The presence of detectable expression levels of the 80-gene panel in benign lesions suggest these genes may have been activated early in tumor growth preceding malignancy. While more studies are needed to investigate whether the 80-gene panel has utility for detection of pre-cancer lesions, the current results provide evidence that the overexpression of the novel panel of 80 genes in tumor samples above background levels in healthy blood samples is reproducible. The results also show that both Affymetrix and NanoString platform results were consistent, showing overexpression of the 80 genes in tumor samples in comparison to healthy donors’ blood samples.

### 2.5. Experimental Validation-Spiking Experiments

**Figure 3:**
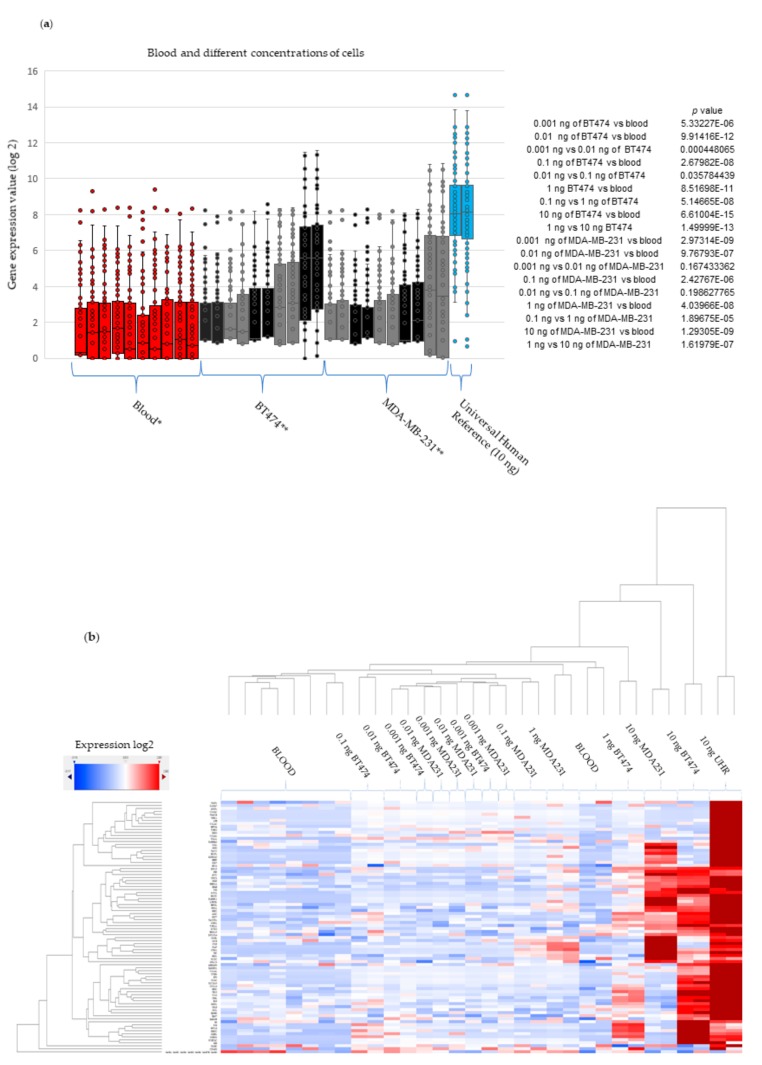
Experimental analysis with different concentrations of RNA from two breast cell lines diluted in pooled RNA from individuals with non-cancerous conditions. (**a**) Boxplots of the expression levels across the different dilution points. The boxplots show the expression levels for each cell analyzed (in duplicate), in order of increasing concentration, when mixed to a pool of 100 ng of blood RNA from cases with non-cancer conditions. It is possible to note that expression levels became expressive, when compared to blood, from 1 ng of RNA from the different lineages of breast cancer cells. (**b**) Heatmaps showing the expression levels across the different dilution points, showing expression distribution for each specimen analyzed. It is possible to note the increase in color intensity with increasing RNA concentration of the two breast cell lines tested in the pool of blood RNA from cases with conditions other than cancer.

The expected detection threshold of the NanoString nCounter platform is 100 ng of RNA. We designed an experimental analysis with different concentrations (see Methods Section) of RNA from two cell lines (MDA-MB-231 and BT-474) diluted in pooled RNA (10 buffy coats) from individuals with non-cancerous conditions to test the hypothetical ability to detect CTCs among the global blood cells. These cell lines were selected because they cover two distinct molecular types of breast cancer (triple negative and luminal B type). Figure 3a,b shows the expression levels in boxplots (with *p*-values) and a heatmap, respectively. In both analyses, the expression levels of the 80 genes were mainly visible from 1 ng and 10 ng of RNA from the cell lines, when taken in consideration that in the other analyzes, the log 2 expression range for the blood was below 4. Note that from 1 ng and 10 ng, the interquartile ranges of log2 expression ranged from 4 to 6, respectively. However, the statistical analyses revealed that all the dilutions were significantly different from blood, demonstrating that the presence of 1 CTC in the bloodstream can be detected by 80 novel biomarkers. The results suggest the utility of the 80 genes as biomarkers for CTC. More studies will be carried out in order to validate the biomarkers described here in larger CTC datasets.

### 2.6. External Validation in Eight Cancer Types

The panel is not intended to describe differences between solid tumors, but rather to identify a panel of biomarkers with differential gene expression between health blood samples and multiple tumor types. Then, to test the expression of the 80 genes in multiple cancer types beyond breast cancer, we analyzed the gene expression profiles in 17 different Affymetrix gene chip datasets representing 8 cancer types (including breast, colon, gastric, leukemia, lung, ovarian, pancreatic, and prostate), 1 dataset of 51 different breast cell lines (E-TABM-157), and 1 dataset of blood samples from both breast cancer patients and healthy controls. The data were quantile normalized within each study.

**Figure 4:**
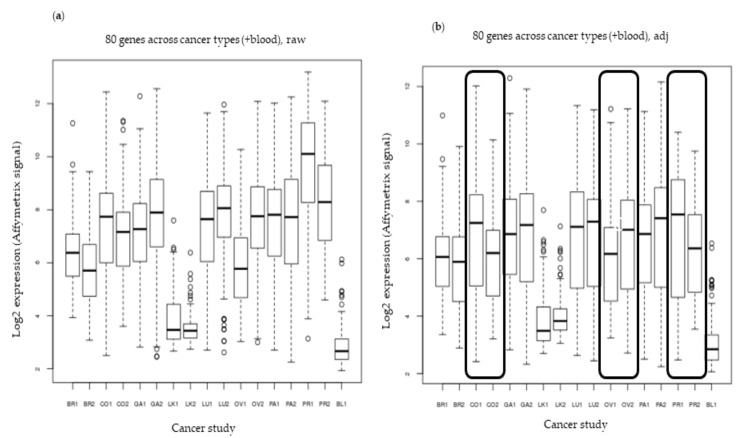
Evaluation of expression of the 80 genes in 17 different datasets including 8 different types of cancer. (**a**) Boxplots of the average expression levels of the 80 genes across 17 expression sets without batch correction. (**b**) Boxplots of the average expression levels across 17 expression sets with batch correction. The medians and interquartile ranges of expression in colon, ovarian, and prostate studies, after batch correction, showed differences among the pairs of the same tissue. These differences are shown by the frames.

Boxplots of the average expression levels across the 17 expression sets show that batch correction was needed (Figure 4a). In order to account for the batch effect, a simple protocol was implemented by finding the average expression of all 22,277 genes in each data set, and using a scale factor correction so that the 17 averages would all be equal. An Excel file of all the averaged values before and after batch correction was created to perform these calculations. 

Furthermore, as indicated in the previous figure, the expression mean of the 80 genes (see Methods Section) for each study was variable, but it is clear that these genes were expressed in higher levels in solid tumors than in leukemia and blood from healthy subjects, whether or not batch correction was performed (Figure 4a,b).

### 2.7. Matched Samples—Tumor Versus Blood

**Figure 5:**
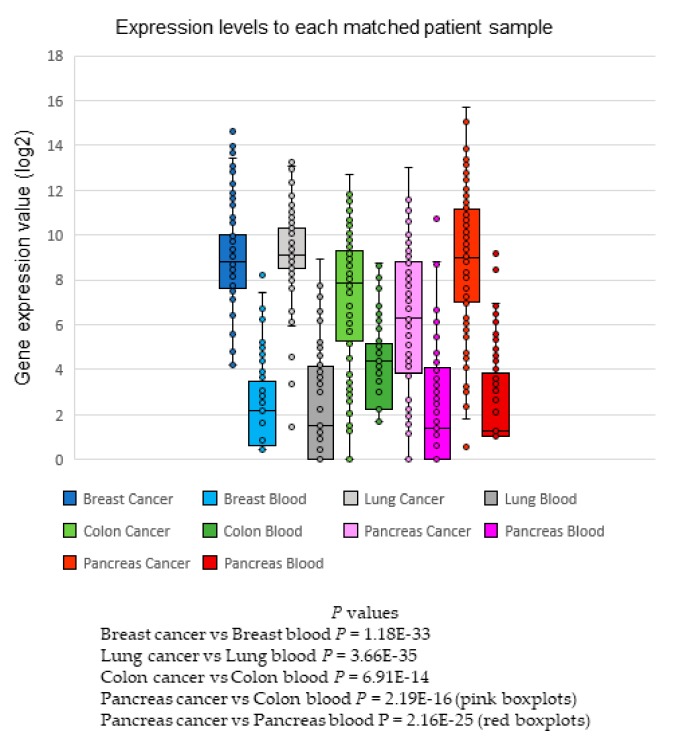
Expression of the 80 genes in 4 different malignant tumors with the blood pair. Boxplots show the expression of the 80 genes in 4 different malignant tumors with the blood pair, including breast, lung, two cases of pancreatic cancer, and colon cancer. The means of expression (log2) for tumors are visible above 8–10 (except for the two cases of pancreatic cancer), and below 4 for blood samples (except for one case of pancreatic cancer, shown as the pink boxplot).

Furthermore, five different tumor and blood pairs from patients with breast, lung, colon, and pancreatic cancer were evaluated for comparison of expression levels of the 80 gene panel in the custom-designed NanoString assay. Supplementary Materials Table S4 shows the histological data of cases analyzed. Figure 5 and Supplementary Materials Figure S1 show the boxplots and heatmap, respectively, of the expression levels for each sample pair evaluated. Note that in all tumors, the interquartile ranges of log2 expression ranged from 4 to 10, whereas the blood interquartile ranges reached their maximum at 4 (background), except for colon cancer patients. In all comparisons, the bloods versus tumor expression differences were statistically significant. This is relevant because it shows the biomarkers were dominant in tumor samples; therefore, any signal found in blood should come from tumor cells. The expression ratio from tumor to blood was indicative of far fewer CTCs compared to the primary tumor. Further investigation will be needed to compare large datasets of healthy samples with patient blood samples to discriminate CTCs. Nevertheless, these results confirm the dominant expression of the 80 genes in solid tumors.

### 2.8. External Validation in CTCs

**Figure 6:**
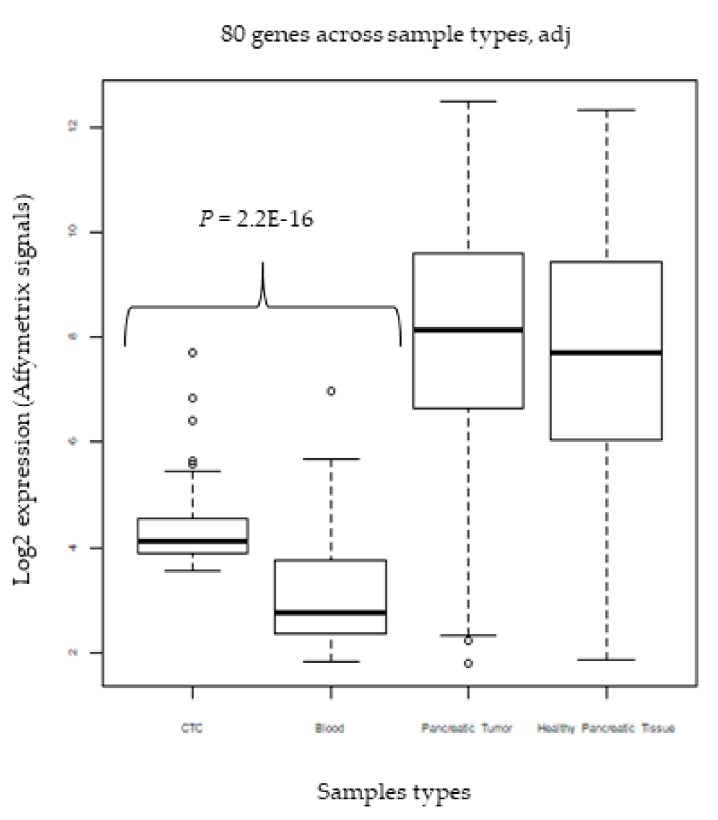
Boxplots showing the average expression levels of the 80 genes for each specimen group. Levels of expression of the 80 genes in the analyzed species of the study GSE18670, which consisted of CTCs, hematological cells, original tumor, and non-tumor pancreatic control tissue of patients with pancreatic ductal adenocarcinoma. High levels of expression, greater than 6 (log2), can be noted for tumor and non-tumor tissue. For CTCs, this level of expression was higher than 4, whereas for blood, these levels were less than 4.

To evaluate the ability of the novel blood biomarker panel of 80 genes to detect CTCs from patients’ blood samples, we analyzed an independent data set of CTCs from patients with pancreatic tumors [27] (see Methods Section). In this study, CTCs were enriched using density centrifugation and isolated using fluorescence-activated cell sorting (FACS) with a negative depletion procedure, combining gates containing CD (cluster of differentiation) 45 negative and CD34 negative cells in addition to 7-amino-actinomycin D viability staining to exclude all hematological and non-viable cells. From this study, we analyzed the levels of expression of the 80-gene panel in the CTCs, pancreatic tumor, tissue adjacent to the tumor, and the blood of the same patients in the study (six patients), totaling 24 samples. Figure 6 shows the expression boxplots for each specimen evaluated and compared to blood expression levels. The largest expression difference was between the pancreatic tumors and adjacent tissue in relation to blood and CTCs. However, using Student’s *t*-test, the difference in expression between blood and CTCs was statistically significant, with a *p*-value of 2.2e-16. The results suggest that the novel gene panel can be used to detect CTCs in patients’ blood samples, but the small sample size limits the conclusions from this experiment until further validation in a larger patient cohort.

## 3. Discussion

The detection of CTCs by epithelial markers are traditionally due to the epithelial origin of solid tumors. However, it has been shown that these cells phenotypically lose the epithelial expression profile when they undergo the epithelial–mesenchymal transition (EMT) that is characteristic of invasive and metastatic stages of cancer. During this process, they recruit and express factors that will favor their mobility and escape the reactions of the immune system [28,29,30]. For this reason, isolation methods such as CellSearch, which exclusively uses epithelial markers, would not detect cells with metastatic potential [31]. In the same scenario, the only molecular test on the market for the detection of specific CTC transcripts in blood, AdnaTest (QIAGEN), has shown sensitivity and specificity similar to CellSearch [32]. However, when used in patients under neoadjuvant chemotherapy, followed by surgery and adjuvant chemotherapy, it has revealed cases of false negatives. This happens because the test generally uses three genes for CTCs detection, and these genes could change their expression profile with tumor evolution [33].

We describe here a panel of biomarkers capable of discriminating breast cancer and six other solid tumors from healthy blood samples (Figure 4a,b). To reach the final list of genes, 859 samples were analyzed at the discovery stage, consisting of 417 mammary tumors (different histopathologies), 41 breast lines (luminal, basal, HER2, and non-tumoral), and 401 control samples (individuals with conditions other than cancer). We were able to exclude the large background of immune cell gene expression in the blood and then compared the blood samples to the datasets of tumors and mammary cells. Later, the 80 selected genes were reassessed in data sets of different tumors and CTCs, in addition to being validated in samples of malignant and benign lesions, and breast cell lines were analyzed on the NanoString platform. As demonstrated in the validation results (Figure 2 and Figure 3), it is possible to observe the high expression of these genes in the tumors compared to blood expression levels.

Among the genes tested, we highlighted the classical epithelial markers for the detection of CTCs, such as *KRT7* (keratin 7), *KRT19* (keratin 19), and *EpCAM*. Furthermore, we highlighted four genes involved in the synthesis of collagen, cellular plasticity, and EMT: *COL1A* (collagen, type I, alpha 1), *COL1A2* (collagen, type I, alpha 2), *COL5A1* (collagen, type V, alpha 1)*,* and *COL5A2* (collagen, type V, alpha 2). These genes were described in their discovery as up-regulated in connective tissues such as osseous tissue and dermis. Mutations in these genes are associated with osteogenesis imperfecta types I–IV, Ehlers–Danlos syndrome type VIIB, recessive Ehlers–Danlos syndrome classical type, idiopathic osteoporosis, and atypical Marfan syndrome [34]. More recently, they have been described in malignant transformation of gastric tissue and lung adenocarcinoma metastases [35,36].

The mechanisms involved in mobility and plasticity that allow CTCs to initiate a secondary tumor site are still an open question. For a long time, the metastatic process was believed to be exclusive to advanced tumors, but that does not explain why patients with in situ and early tumors would sometimes experience distant metastases [11,12,13,14]. Some of these questions have been answered by the first studies that described genetic signatures distinguishing patients with early breast tumors from disseminated tumor cells in the bone marrow. These studies demonstrated that the process of cellular dissemination still begins in the formation of a primary site. Among the genes involved in these mechanisms are those involved in the functions of extracellular matrix remodeling, adhesion, cytoskeleton plasticity, regulation of transcription, ATP binding, and signal transduction [37,38]. Accordingly, we describe here genes involved in cell motility and plasticity, *CDH1* (E-cadherin), *CDH11* (cadherin-11), *ENAH* (enabled homolog), *FNBP1L* (formin binding protein 1-like), and *GJA1* (gap junction protein); cell migration, *NRP1* (neuropolin-1), *PLAT* (plasminogen activator), *PLEKHC1* (fermitin family), and *TIMP3* (metallopeptidase inhibitor 3); and cell cycle and transcriptional factors synthesis, *DKK3* (dickkopf homolog), *ECT2* (epithelial cell transforming sequence 2 oncogene), *GHR* (growth hormone receptor), *GINS1* (gins complex subunit 1), *GPR125* (G protein-coupled receptor 125), *NEK2* (never in mitosis gene a-related kinase 2), *VGLL1* (vestigid like 1), *TFAP2P* (transcription factor AP-2 beta), and *TTK* (ttk protein kinase).

Smirnov et al. [24] have described 25 candidate genes for discriminating CTCs in the bloodstream. However, when evaluated in CTCs from patients with metastatic breast (*n = 13*), prostate (*n* = 31), and colon (*n* = 30) cancers, the results revealed 9 of these 25 genes up-regulated in normal blood. Moreover, some of these genes presented clusters of specificity for the respective tumors analyzed, not covering the potential of being used as biomarkers of CTCs isolation for multiple tumors. Four of these genes are common to the panel described herein: *KRT19*, *ASGR2* (asialoglycoprotetin receptor 2), *SCGB2A2* (mammoglobin 1), and *SLC2A10* (glucose transporter 10). Furthermore, Gorges et al. [25], analyzing CTCs of patients with breast cancer and metastatic prostate cancer using single-cell transcriptional analysis technology, revealed genes in common with those described here. Among them we can cite the high expression of *CDH1*, *COL1A2*, *COL1A5*, *EpCAM*, and *KRT19*. Along with Lang et al. [39] who analyzed CTCs from patients with metastatic breast cancer, we confirm here a transcriptional phenotype similar to aggressive tumors, with low expression of genes involved in cell apoptosis, absence of immune signals, low ribosomal activity, and high expression of genes involved in mobility and cell adhesion. These factors confirm the hypothesis that CTCs during the circulatory stage in the blood present a phenotype of dormancy, unperceived to the immune system against a metastatic process [40].

The identification of a novel biomarker for identification of circulating tumor cells (CTC) in blood will likely have a wide impact on cancer understating and patient care by supplementing existing methods and enhancing novel technologies. Furthermore, advances in CTC detection may open new avenues for understanding tumor cell dissemination and metastasis. The current 80-gene panel expands the number of candidate biomarkers for screening, detecting, and monitoring the treatment of not only advanced cancers, but also early tumors with known site of origin, as it encompasses genes with different functions and demonstrated relevant expression in different solid tumors in our study. Furthermore, these biomarkers may contribute to the emerging advances in liquid biopsy as the initial identification of these transcripts in circulation, as circulating free RNA or encapsulated by tumor cell-derived exosomes, further identified by associated microRNAs [41]. Although we present experimental validation of the differential gene expression of the 80-gene panel in in breast tissue samples and blood—spiking experiments and matched samples showing significant expression for four different tumors (breast, lung, colon, and pancreatic cancer)—in order to develop these markers as clinical diagnostics for adoption, large scale prospective clinical studies including thousands of patients will be required. Considering from the discovery to the phase of clinical application, further experimental validations should be performed on a larger cohort of patients to provide statistical power, as well as on non-breast tumor types, as performed herein. Another area of development for the 80-gene panel is to study targeted panels for specific tumor types for screening tumors of unknown origin sites [42]. In addition, isolation and enrichment methodologies should be compared to identify the genes with the highest sensitivity and specificity among the 80 genes. Initially, we prioritized membrane protein coding genes as a strategy for identifying the best candidate genes for detecting CTCs, with this possibly being used as a methodology for their isolation [43]. This opens avenues for extending and applying these genes to simple technologies such as cytometry, adhesion chips, and magnetic beads. Thus, further research will be needed to validate tumor-specific putative markers for independent analysis.

## 4. Methods

### 4.1. Discovery Set

Genome-wide expression microarrays (Affymetrix HG-U133 plus 2.0) were used to compare breast tumor tissue biopsies and breast cancer cell lines with blood samples from patients with conditions other than cancer. Genes were selected that were expressed at high levels in breast cancer samples but not expressed above the microarray detection background in blood samples. A total of 859 samples were used in this comparison, including 417 breast tumor tissue biopsies (GSE2034 and [44]), 41 breast cancer cell lines (GSE16795 and GSE8096, [45]), and 401 blood samples (GSE5418, GSE12288, GSE1343, GSE3846, GSE6269). Blood samples were obtained from five different publicly available datasets, and were collected from individuals with conditions other than cancer, including bacterial infections, cardiac conditions, and following the consumption of various beverages. Eighty unique genes (85 Affymetrix probesets) were selected that were expressed at background levels in controls and high levels in breast tumors. A detailed outline of the gene selection procedure used here is shown (Figure 1a).

### 4.2. External Validation 1

Expression levels of the 80 genes were studied in independent sets of breast tumor samples (GSE20194, GSE18864) and normalized to the median of an independent set of control samples (GSE19314). The total of 348 breast tumor samples were normalized to blood samples obtained from 66 patients with conditions other than cancer. The breast tumor samples included in this set included 278 breast cancer biopsies obtained by fine needle aspiration (80% breast tumor cells) (GSE20194), and 70 breast cancer biopsies (GSE18864) that were prepared by micro-dissection to include only breast tumor cells. 

In the second analysis, we compared the same tumor samples with two PBMC (GSE11281 and GSE11881) datasets from patients with non-cancerous conditions, totaling 26 new samples.

### 4.3. External Validation 2

The expression levels of the 80 genes were studied in 39 breast cancer cell lines (GSE16795), and were normalized to 66 non-cancer blood samples (GSE19314). The cell line dataset was the same as that used for the original selection procedure.

### 4.4. External Validation 3

Then, to evaluate the potential of these biomarkers in the discrimination of other types of cancer beyond breast cancer, the 80 genes were analyzed in 17 different datasets in total from 8 cancer types (breast, colon, lung, ovarian, prostate, pancreatic, gastric cancers, and leukemia (GSE25055, GSE39582, GSE68468, GSE13911, GSE54129, GSE13159, GSE14471, GSE19188, GSE30219, GSE26712, GSE9891, GSE15471, GSE16515, GSE17951, GSE8218)), and one dataset of 51 different breast cell lines (E-TABM-157), compared to one dataset of human peripheral blood mononuclear cells (PBMCs) from breast cancer patients, patients with benign breast abnormalities, healthy cancer-free individuals, and patients with other types of cancer (gastrointestinal and brain cancers) (GSE27562). Some samples were removed if they were in the wrong tissue; for example, CO2 (colon) contained some normal liver and prostate cancer samples. One of the samples for LK2 (Leukemia 2) had a corrupted CEL file and could not be used (GSM361532). The leukemia LK1 study (GSE13159) included a total of 2096 samples from many different kinds of leukemia, but we restricted our analyses to acute myeloid leukemia (AML) and its genetic subtypes. As a result, the numbers of samples listed are the ones actually used in our analyses, which were sometimes lower than the total number of samples listed in Gene Expression Omnibus (GEO) or ArrayExpress. There was a total of 3601 samples analyzed.

### 4.5. External Validation 4

An independent external validation was performed to evaluate the potential of the 80 genes in CTC discrimination. Only one NCBI (National Center for Biotechnology Information) Gene Expression Omnibus (GEO) study was found by analyzing CTCs from the Affymetrix platform (GSE18670) including six samples. The expression of the 80 genes was compared among specimens evaluated in this dataset, which consisted of CTCs, hematological cells, original tumor, and adjacent-pancreatic tissue of patients with pancreatic ductal adenocarcinoma (totaling 24 samples).

### 4.6. Affymetrix Data analysis 

All databases (Affymetrix) and the number of cases used for discovery and external validations are described in Table 1. For each of the studies, the CEL files (Affymetrix file extension) were read into R using the Bioconductor package “oligo” and the annotation packages for HG-U133A and U133 Plus 2.0 arrays (pd.hg.u133a, pd.hg.u133.plus.2). The genes were quantile-normalized within each study using RMA, and the sample information files were attached to the expression data in a Bioconductor ExpressionSet (eset) data structure, using the R script create_esets.R.

In order to account for the batch effect, a simple protocol was implemented by finding the average expression of all 22K genes in each data set, and using a scale factor correction so that the 17 averages (15 cancer datasets, 1 dataset of 51 different breast cell lines (E-TABM-157 and 1 blood dataset)) would all be equal. An Excel file of all the averaged values before and after batch correction was created to do these calculations. Four clinical biomarkers for breast cancer were included in the analysis as controls: estrogen receptor (*ESR1*), progesterone receptor (*PGR*), human epidermal growth factor receptor 2 (*ERBB2*), and prolactin receptor (*PRLR*).

### 4.7. Experimental Validation 

#### 4.7.1. NanoString Assays 

For quantitative assessment of expression of 80 genes, we designed a custom nCounter assay (NanoString Technologies, Seattle, WA). In addition to the 80 genes, there were 7 housekeeping genes, 5 positive control genes, 8 negative control genes, and 4 breast cancer clinical biomarkers (*ESR1*, *HER*, *PGR*, and *PRLR*) included in the same panel. These genes were included because they are traditional biomarkers for mammary carcinogenesis. The full list with the 104 genes can be found in Supplementary Materials Table S5.

#### 4.7.2. Breast Tissue Samples

Patients were recruited from 2015 to 2016 at the Mauricio Magalhães Costa Clinic, Américas Barra Medical City, in the city of Rio de Janeiro, Brazil. The study was conducted according to the guidelines of the Declaration of Helsinki, and all patients provided written informed consent. This study was approved by the ethics committee of Rio de Janeiro State University Hospital (Identification code number: 43560115.5.0000.5259, date: 06/10/2015). Forty-four formalin-fixed paraffin-embedded (FFPE) biological samples were analyzed, including malignant (*n* = 36) and benign breast lesions (*n* = 8) (Supplementary Materials Table S2). RNA of each specimen was extracted from two sections of 10 μM using the RNeasy FFPE kit (QIAGEN, Hilden, Germany), according to the manufacturer’s protocol with modifications. The incubation time with proteinase K suggested for 15 min was extended to 30 min.

#### 4.7.3. Blood Samples

Blood samples were obtained from the University of Connecticut’s tissue bank. A total of 32 buffy coats from women with conditions other than cancer were included in the study (Table S3). The buffy coats were isolated from 10 mL of whole blood. RNA of each specimen was extracted using the NucleoSpin RNA Blood Kit (Macherey-Nagel, Düren, Germany), according to the manufacturer’s protocol.

#### 4.7.4. Breast Cell Lines 

The BT-474, Hs578T, MDA-MB-231, MCF-7, MCF-10A, MCF-12A, T47D, and ZR-75B cell lines were used in this study. These cells were obtained from the American Type Culture Collection (ATCC) (Manassas, VA, USA) in 2017, except for MDA-MB-231, MCF7, and MCF10, which were donated from the Department of Cell Biology, University of Connecticut, but originally obtained from the ATCC (Manassas, VA, USA) in 2015. Hs578T and MDA-MB-231 were cultured in Dulbecco’s modified Eagle’s medium (DMEM) (Gibco-11965, Grand Island, NY, USA), supplemented with 10% fetal bovine serum (FBS), 2 mM of L-glutamine, and antibiotic-antimycotic (1 x) (Gibco, Grand Island, NY, USA). BT474, MCF-7, T47D, and ZR-75B were cultured in Dulbecco’s modified Eagle’s medium (DMEM), no phenol red (Gibco-21063029, Grand Island, NY, USA), supplemented with 10% FBS, 2 mM of l-glutamine, and antibiotic-antimycotic (1 x) (Gibco, Grand Island, NY, USA). MCF-10A and MCF-12A cells were cultured in MEGM Mammary Epithelial Cell Growth Medium Bullekit (Lonza-CC3150, Portsmouth, NH, USA). The cells were incubated in a standard cell culture incubator (Thermo, Waltham, MA, USA) at 37 °C with 5% CO_2_. All cell lines were tested, authenticated, and declared free from other cell contaminations by short tandem repeat analysis in December 2017.

The RNA of each cell was extracted from 1 × 10^6^ cells, using RNeasy Plus Micro Kit (QIAGEN, Hilden, Germany), according to the manufacturer’s protocol. The cells were automatically counted using TC20 automated cell counter (BioRad, Hercules, CA, USA), according the manufacturer’s protocol.

#### 4.7.5. Spiking Experiments

The BTB-474 and MDA-MB-231 human breast cancer cell lines were used to test the sensitivity and specificity of the test in the detection of CTCs.

To test the feasibility of the experiment, the RNA from each cell line was diluted to different concentrations: 10, 1, 0.1, 0.01, and 0.001 ng/µL, equivalent to 10^3^, 10^2^,10, 1, and 0.1 CTCs, respectively [46]. These dilutions were mixed with an RNA pool (100 ng/µL, equivalent to 10^4^ cells) of blood from 10 women with conditions other than cancer (randomly selected samples, see above in Section 4.7.3). All assays were performed in technical duplicates. The Universal Human Reference RNAs (UHR) (Agilent technologies, TX, USA) at a concentration of 10 ng/μL and RNase-free water (QIAGEN, Hilden, Germany) were used as a positive and negative control for the assays, respectively.

#### 4.7.6. Matched Samples—Tumor versus Blood

Five different tumor and blood pairs from patients with breast, lung, colon, and pancreatic cancer were evaluated for comparison of expression levels of the 80-gene panel in the custom-designed NanoString assay. Tumor and blood samples were obtained from the University of Connecticut’s tissue bank. RNA of each tumor specimen was extracted from two sections of 10 μM using the RNeasy FFPE kit (QIAGEN, Hilden, Germany), according to the manufacturer’s protocol with modifications. The incubation time with proteinase K suggested for 15 min was extended to 30 min. The buffy coats were isolated from 10 mL of whole blood. RNA of each specimen was extracted using the NucleoSpin RNA Blood Kit (Macherey-Nagel, Düren, Germany), according to the manufacturer’s protocol.

#### 4.7.7. Quantification and Quality (QC) Metrics

All the samples had quantification and quality (QC) results through the Nanodrop 1000 (Thermo Scientific, Wilmington, DE) and Qubit RNA Assay Kit (Invitrogen, Carlsbad, CA, USA) according to the manufacturer’s protocol.

### 4.8. NanoString Protocol

#### 4.8.1. Sample Preparation and Hybridization 

The appropriate mass of sample was prepared according to the NanoString protocol. A total of 5 µL of each sample was mixed with 8 µL of the hybridization cocktail, containing the reporter codeset and the hybridization buffer. A total of 2 µL of the capture codeset was added, and the solution was mixed and spun down. The UHR (Agilent technologies, Wyldwood, TX, USA) and RNase-free water (QIAGEN, Hilden, Germany) were used as positive and negative controls for the assays, respectively. It was placed in a 65 °C thermocycler (Veriti Thermal Cycler, Applied Biosystems, Foster City, CA, USA) for 18 h.

#### 4.8.2. Preparation Station and Digital Analyzer 

The samples were transferred to the preparation station with prepared reagent plates and a cartridge. The samples ran with the standard sensitivity for maximum binding to the cartridge. The preparation station ran for approximately 3 h.

The cartridges were transferred to the digital analyzer (NanoString Technologies, Seattle, WA, USA) for analysis. A field of view (FOV) of 280 was used for the cartridges of the project because of expected lower expression levels of the genes of interest. The digital analyzer ran for approximately 2 h and 30 min for each cartridge.

#### 4.8.3. NanoString Custom-Designed Assay Data Analysis 

The raw expression data (RCC files) were normalized using nSolver Analysis software (Version 4.0, NanoString Technologies, Seattle, WA, USA). The normalization was performed according to the manufacturer ’s protocols (nSolver 4.0 User Manual). Briefly, a normalization factor was calculated by obtaining the geometric mean of the positive controls used for each sample and applied to the raw counts of the nCounter output data to eliminate variability that was unrelated to the samples. The resulting data were normalized again with the geometric mean of the housekeeping genes. Normalized data were log_2_-transformed and exported to Microsoft Excel for analysis. The normalized log_2_-transformed mRNA (messenger RNA) expression data were used to create heatmaps. Heatmaps showing high and low expression for each specimen analyzed were created.

Furthermore, in some cases, expression representations were plotted on boxplot model graphs. Boxplot is a method for graphically depicting groups of numerical data through their quartiles. The boxplot has a straight line (whisker) that extends vertically or horizontally from the box, indicating variability outside the upper and lower quartile [47]. Atypical values or outliers can be plotted as individual points. The boxplot is not parametric, showing variation in samples from a statistical population without making any assumption of the underlying statistical distribution. The spaces between the different parts of the box indicate the degree of dispersion, the obliquity in the data, and the outliers. Thus, the boxplots represent as a whole the expression levels of the 80 genes, and their dots represent the expression variability. 

For the spiking experiments, Student’s *t*-test was used to compare single-gene expression levels between blood and the different concentrations of cell lines. All tests were two-sided at the significance level *p* < 0.05.

## 5. Conclusions 

In summary, we describe here a large panel of genes with the potential to detect CTCs present in the bloodstream from multiple tumors. Our study differs from the other panels already described [24,25]. We present a flow chart of external and laboratory validations, totaling 5028 specimens analyzed, and the genes described here can open the way for further investigations involving metastatic processes. Our panel will have the potential for application on other platforms, such as current chip, fluid, and imaging technologies for CTC detection. The technology has been designed for early detection, but can also be applied to monitor cancer progression or treatment response, or for the development of novel diagnostics.

## Figures and Tables

**Table 1:** Datasets used for discovery and external validation of the 80 genes *.

Specification	Accession	Number of Samples
Blood samples from non-breast cancer patients	GSE5418	401
	GSE12288	
	GSE1343	
	GSE3846	
	GSE6269	
Breast cancer biopsies	GSE2034	417
	Hess et al. [44]	
Human breast cell lines	GSE16795	41
	GSE8096	
Whole blood samples from non-breast cancer patients	GSE19314	66
PBMC samples from non-breast cancer patients	GSE11281	26
	GSE11881	
Micro-dissected breast tumor biopsies	GSE18864	70
Fine needle aspirates of breast tumors	GSE20194	278
Eight cancer types (breast, colon, lung, ovarian, prostate, pancreatic, gastric cancers, and leukemia) and 51 different breast cell lines	GSE25055, GSE39582, GSE68468, GSE13911, GSE54129, GSE13159, GSE14471, GSE19188, GSE30219, GSE26712, GSE9891, GSE15471, GSE16515, GSE17951, GSE8218 and E-TABM-157	3601
PBMCs from breast cancer patients, patients with benign breast abnormalities, healthy cancer-free individuals, and patients with other types of cancer	GSE27562	
Circulating tumor cells, hematological cells, original tumor, and adjacent-pancreatic tissue of patients with pancreatic ductal adenocarcinoma	GSE18670	24

PBMC: peripheral blood mononuclear cells (GSE11281, GSE11881, and GSE27562). * The 104 samples remaining to total the 5028 samples analyzed were those from the experimental validation (44 breast cancer lesions, 32 buffy coats, 8 human breast cell lines, 10 samples from spiking experiments, and 10 matched blood and tumor samples).

## Supplementary Materials

Supplementary materials can be found at https://www.mdpi.com/1422-0067/20/19/4894/s1. Figure S1: Heatmap showing the expression levels to each match samples. Table S1: List of genes and the expression data in each specimen analyzed. Table S2: Demographic and histopathological data of the breast samples evaluated. Table S3: List of blood samples analyzed. Table S4: Histopathological data of the matching samples evaluated. Table S5: List of genes selected and validated to NanoString assay.
