# Supplementary material for: A Novel Panel of 80 RNA Biomarkers with Differential Expression in Multiple Human Solid Tumors against Healthy Blood Samples
Source: Int J Mol Sci. 2019 Oct 2;20(19):4894. doi: 10.3390/ijms20194894 (PMC6802086; doi:10.3390/ijms20194894)
Supplement: Supplementary file 1 [file ijms-20-04894-s001.zip › ijms-590000-for proofreading-supplementary/Supplementary Table 4.docx]

| **Sample** | **Histological report** |
| --- | --- |
| BA00464 | Breast tumor - Invasive lobular carcinoma - tubule formation: - nuclear pleomorphism: - mitotic activity: - Nottingham score: score = 3/3 score = 2/3 score =2/3 (7 mitosis/10 hpf) 7 of 9 (grade II of III). pT2 pN0 pMn/a. Estrogen Receptor (Clone 6F11) showed a positive result (8/8 reaction) in the infiltrating lobular carcinoma. Progesterone Receptor (Clone PgR636) showed a positive result (8/8 reaction) in the infiltrating lobular carcinoma. CerbB-2 (Clone EP1045Y) showed a bordeline result (2+/3 reaction). HER2 FISH Analysis by integrated oncology are negative. |
| BA00470 | - Lung adenocarcinoma - Well differentiated - pT3 pN0 pMX |
| BA00473 | Rectum adenocarcinoma pT1 pN0 pMX |
| BA00478 | - Solid pseudopapillary neoplasm of pancreas with neuroendocrine differentiation.  - pT1 pN0 pMX |
| BA00480 | - Tumor Type: Ductal adenocarcinoma  - Histologic Grade: Moderately differentiated  - Tumor Site: Uncinate notch region of head of pancreas.  pTNM: pT3; pN1; pM n/a. Tumor cells are positive for CEA and for Ca19-9. |

**Supplementary Table 4.** List of matching samples evaluated in the nanoString assay
