# Supplementary figures and images for: A Novel Panel of 80 RNA Biomarkers with Differential Expression in Multiple Human Solid Tumors against Healthy Blood Samples
Source: Int J Mol Sci. 2019 Oct 2;20(19):4894. doi: 10.3390/ijms20194894 (PMC6802086; doi:10.3390/ijms20194894)

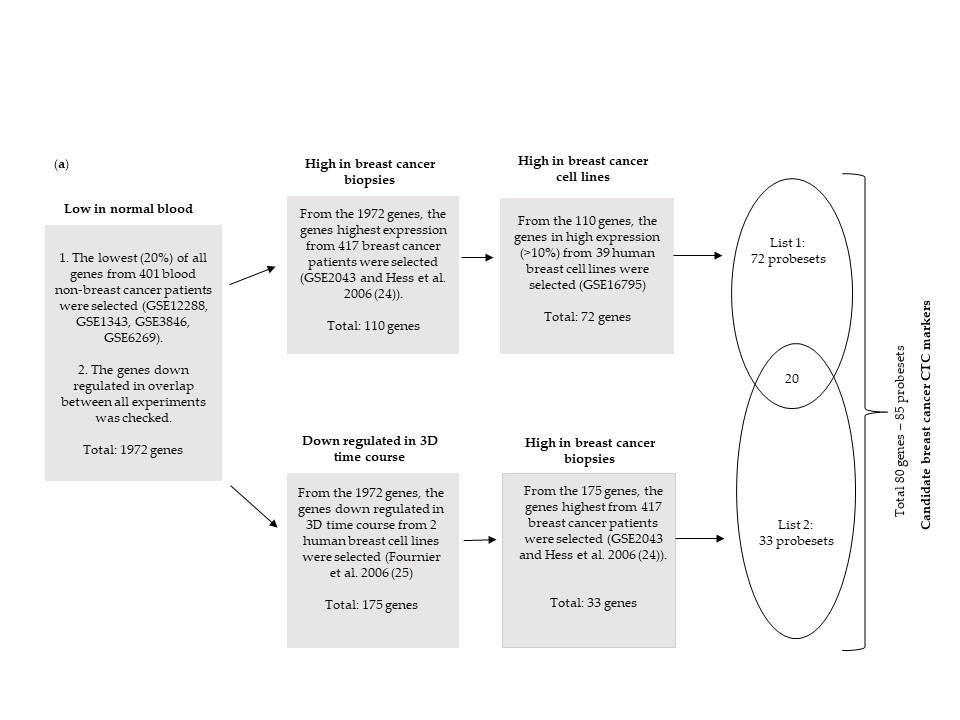

Supplement: Supplementary file 1 [file ijms-20-04894-s001.zip › ijms-590000-for proofreading-supplementary/Figure 1a1.tiff]

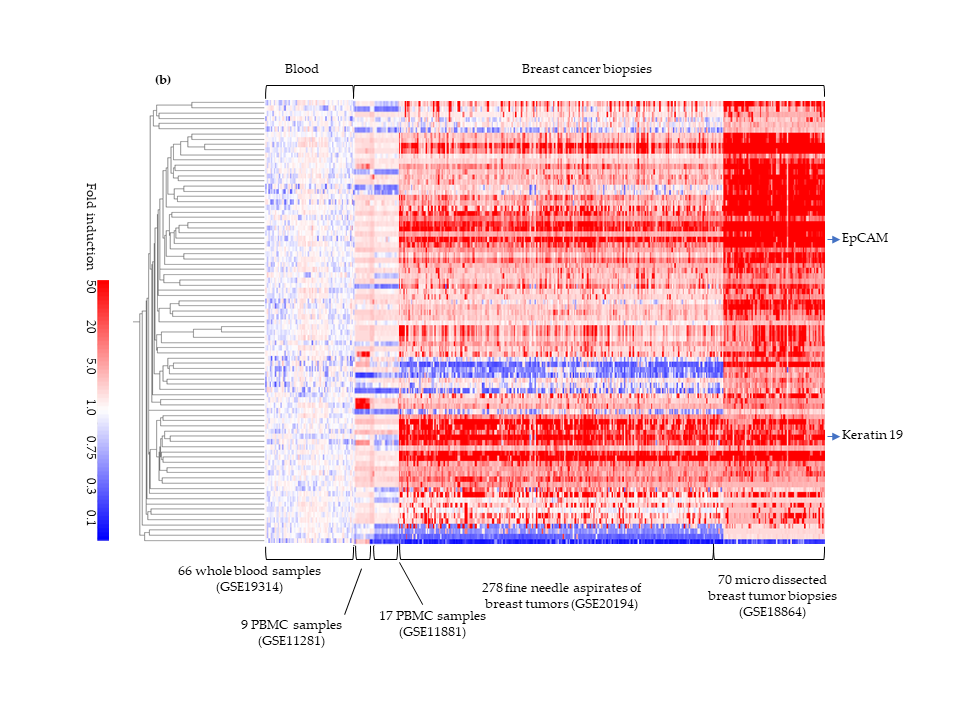

Supplement: Supplementary file 1 [file ijms-20-04894-s001.zip › ijms-590000-for proofreading-supplementary/Figure 1b1.tif]

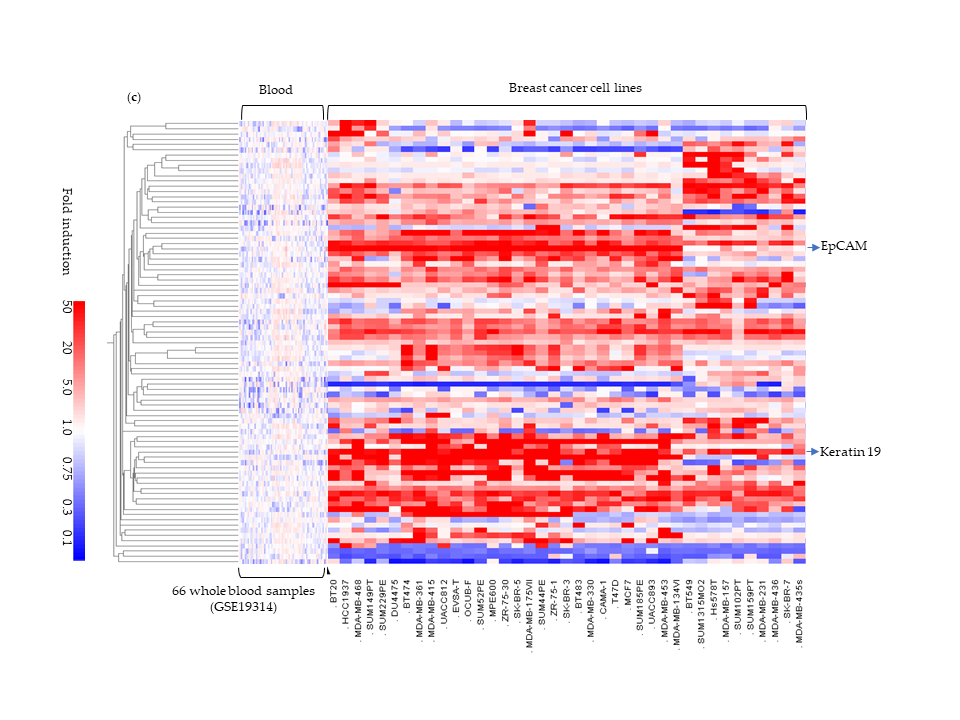

Supplement: Supplementary file 1 [file ijms-20-04894-s001.zip › ijms-590000-for proofreading-supplementary/Figure 1c1.tif]

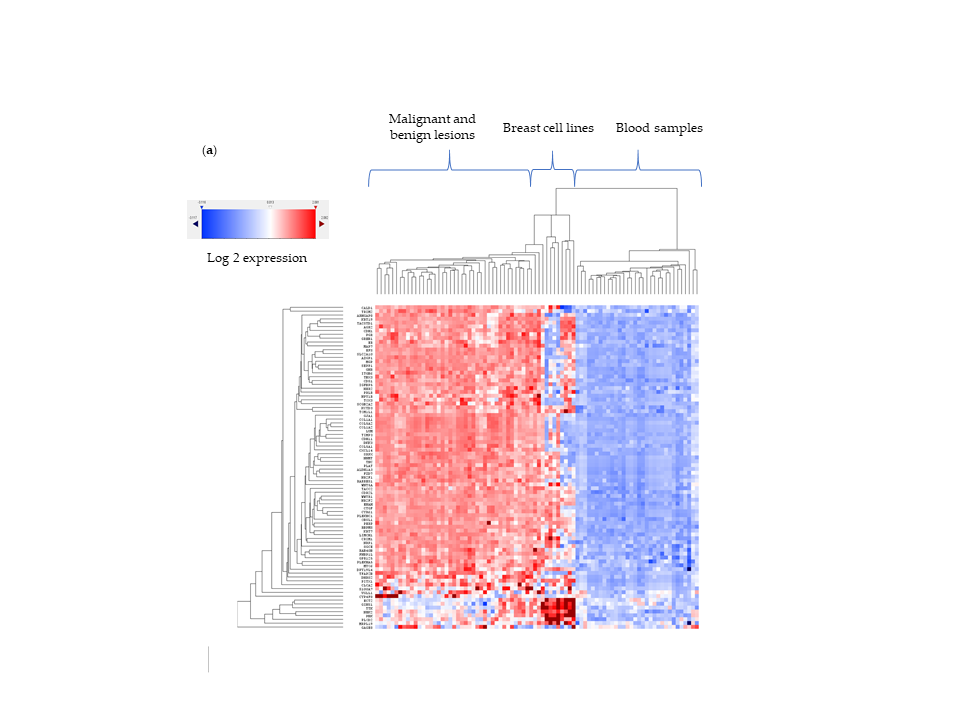

Supplement: Supplementary file 1 [file ijms-20-04894-s001.zip › ijms-590000-for proofreading-supplementary/Figure 2a1.tif]

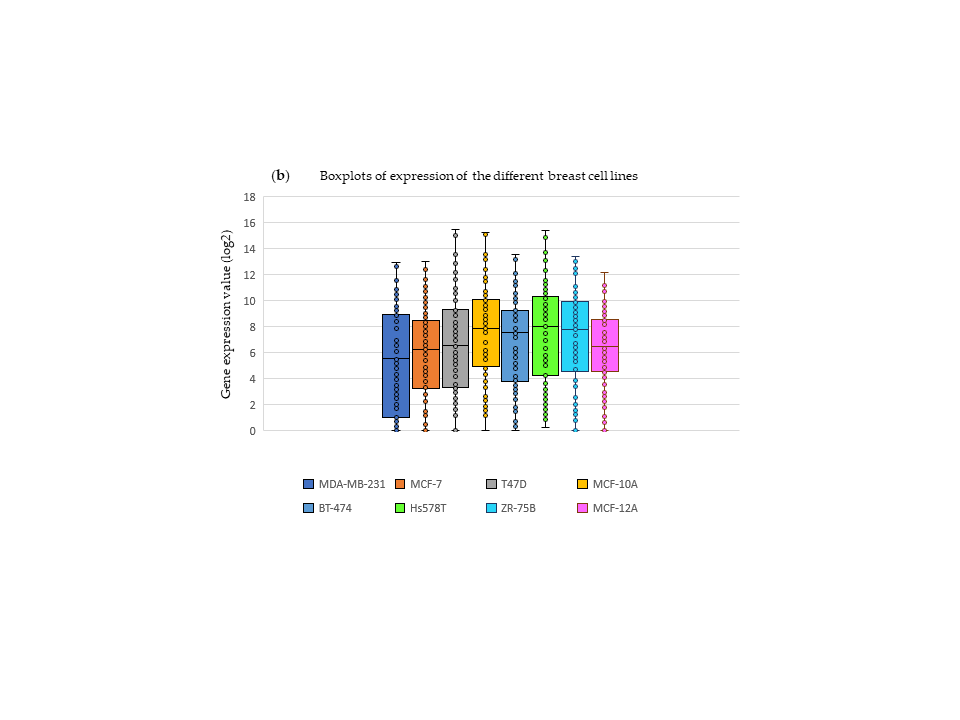

Supplement: Supplementary file 1 [file ijms-20-04894-s001.zip › ijms-590000-for proofreading-supplementary/Figure 2b1.tif]

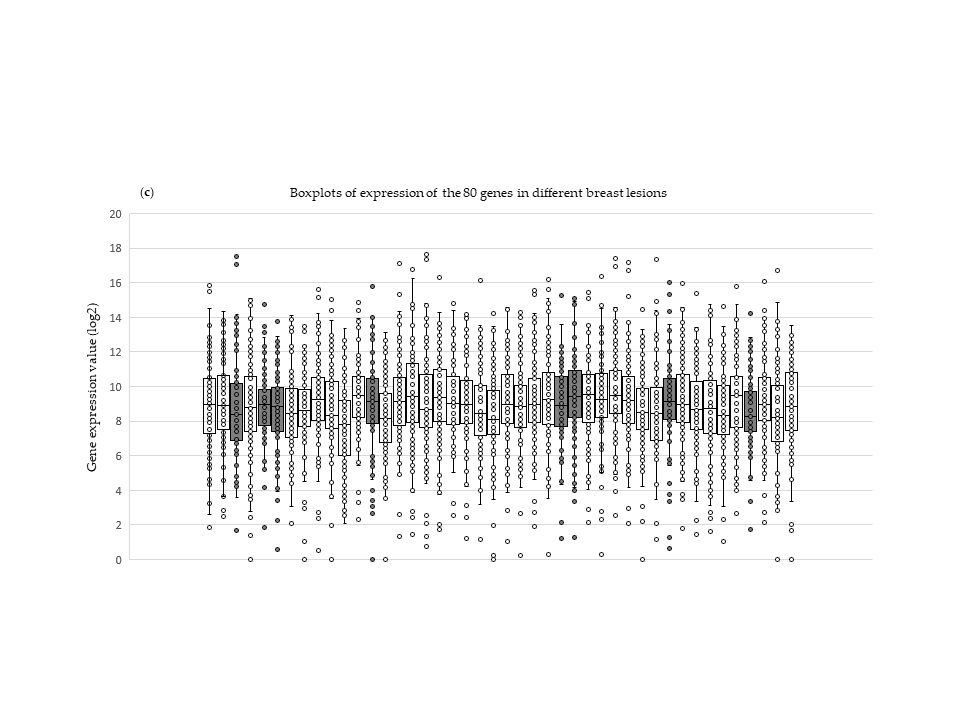

Supplement: Supplementary file 1 [file ijms-20-04894-s001.zip › ijms-590000-for proofreading-supplementary/Figure 2c1.tif]

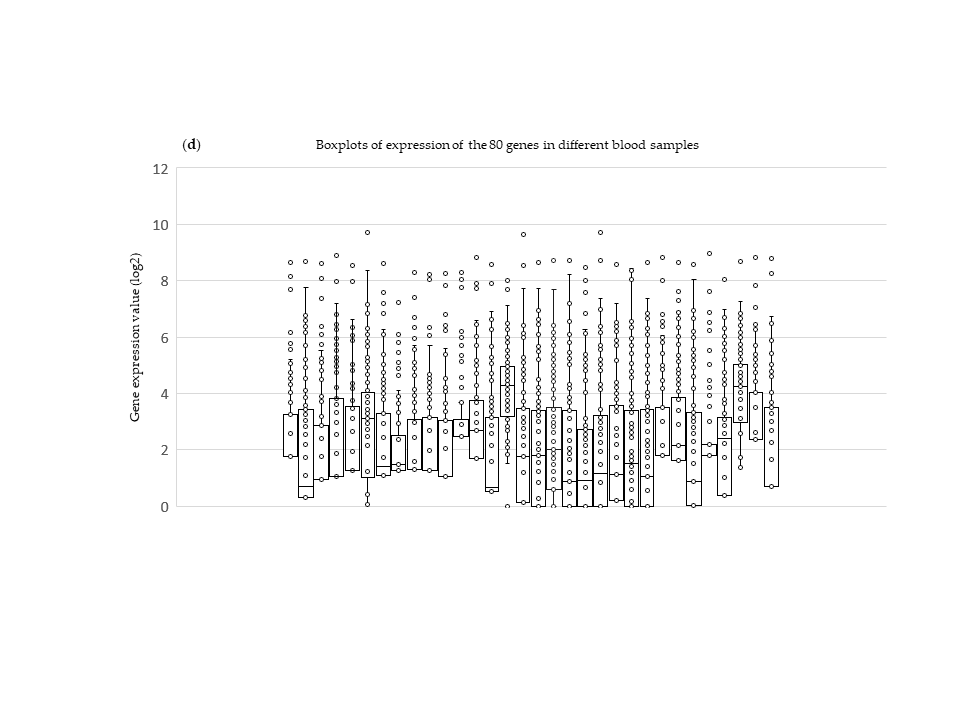

Supplement: Supplementary file 1 [file ijms-20-04894-s001.zip › ijms-590000-for proofreading-supplementary/Figure 2d1.tif]

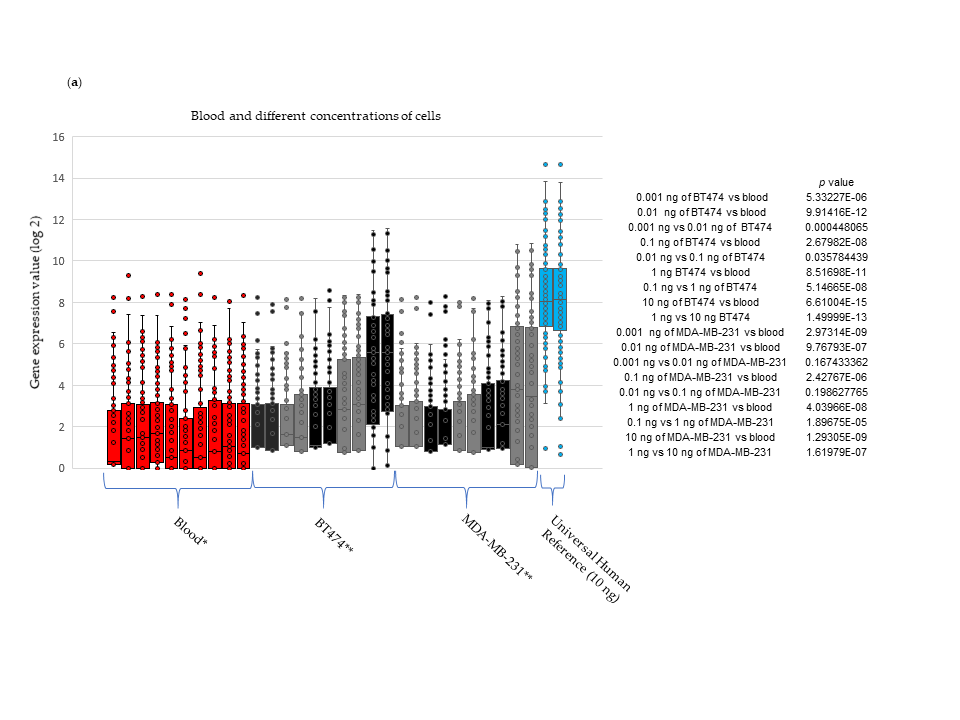

Supplement: Supplementary file 1 [file ijms-20-04894-s001.zip › ijms-590000-for proofreading-supplementary/Figure 3a1.tif]

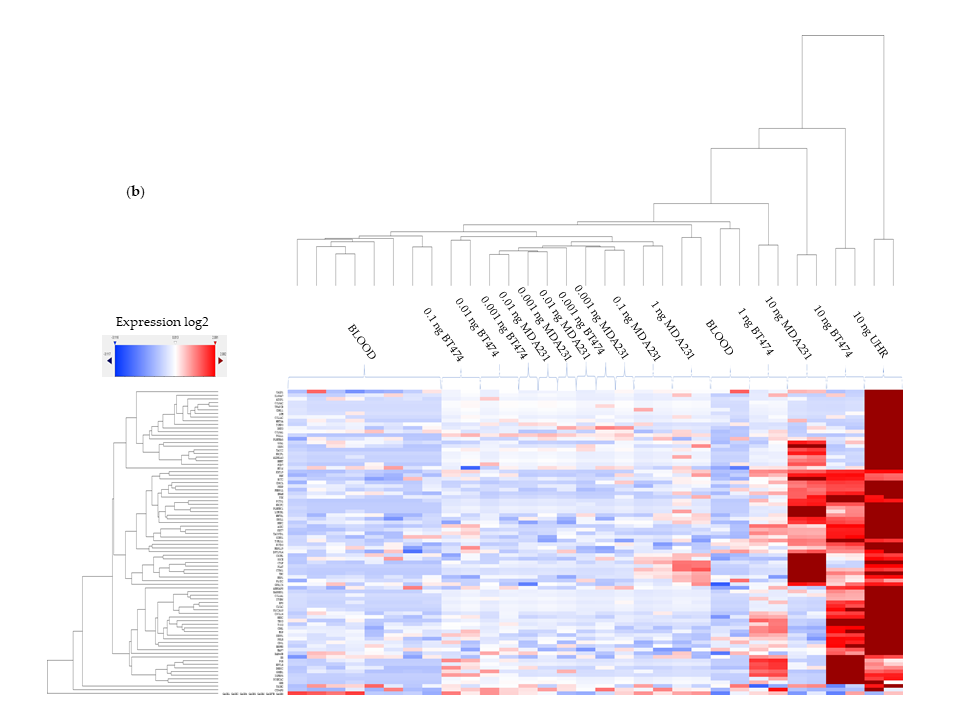

Supplement: Supplementary file 1 [file ijms-20-04894-s001.zip › ijms-590000-for proofreading-supplementary/Figure 3b1.tif]

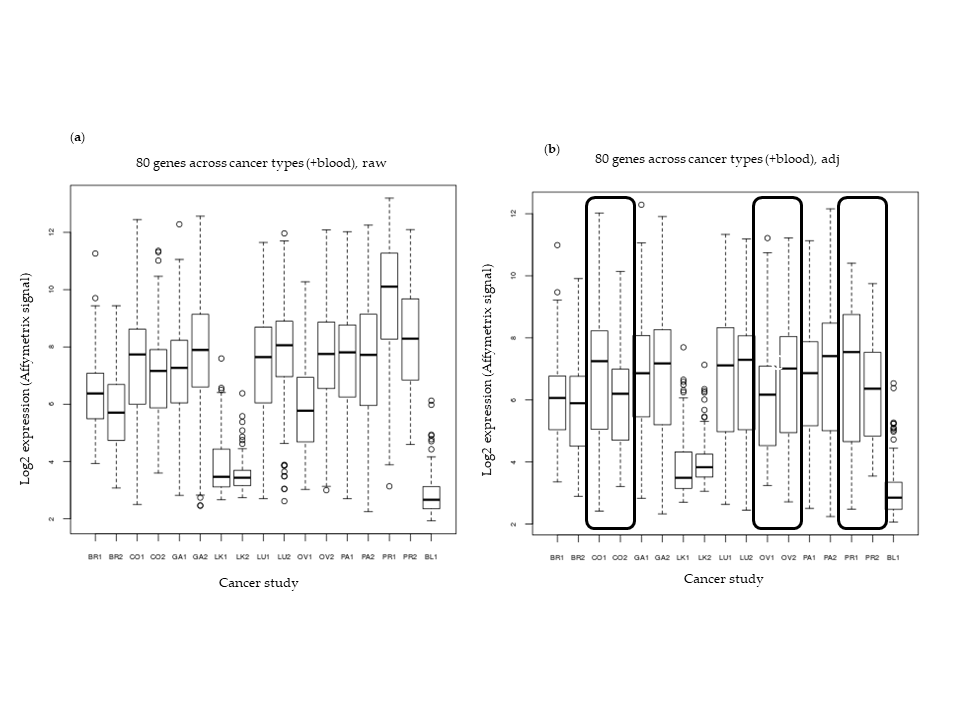

Supplement: Supplementary file 1 [file ijms-20-04894-s001.zip › ijms-590000-for proofreading-supplementary/Figure 4ab1.tif]

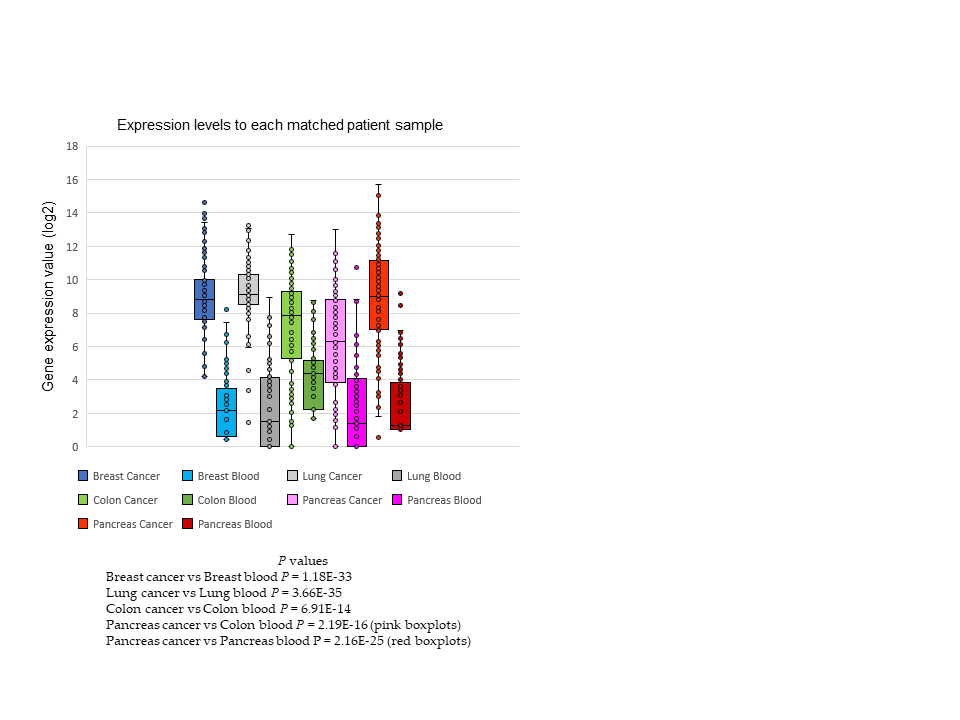

Supplement: Supplementary file 1 [file ijms-20-04894-s001.zip › ijms-590000-for proofreading-supplementary/Figure 5.1.tif]

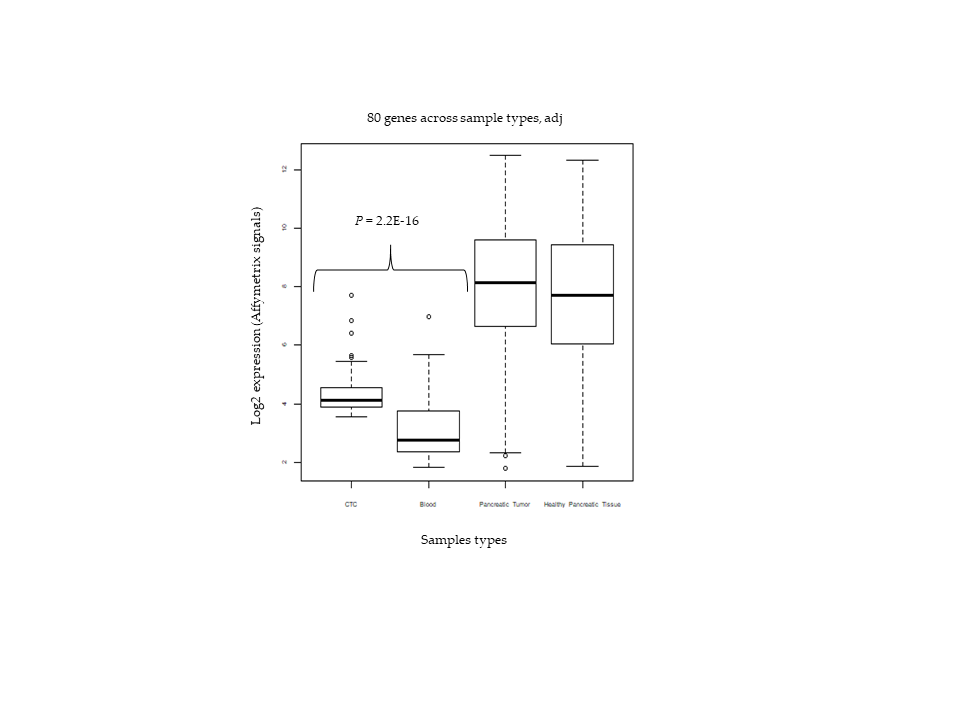

Supplement: Supplementary file 1 [file ijms-20-04894-s001.zip › ijms-590000-for proofreading-supplementary/Figure 6.1.1.tif]

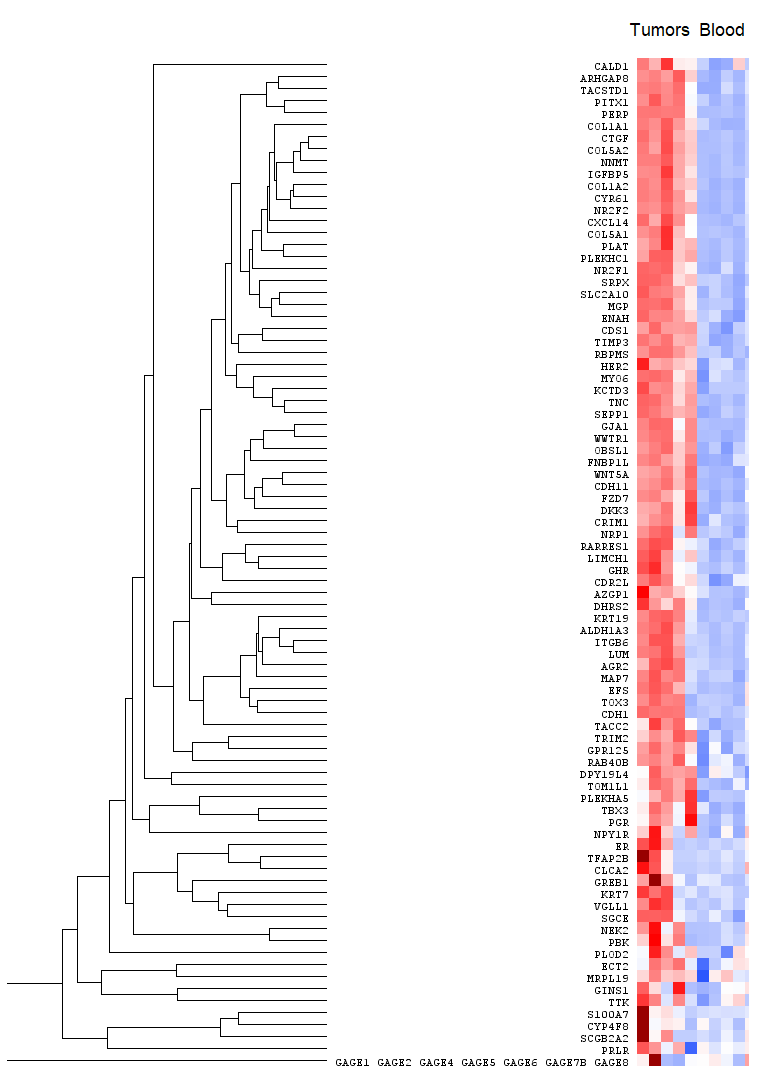

Supplement: Supplementary file 1 [file ijms-20-04894-s001.zip › ijms-590000-for proofreading-supplementary/Suppplementary figure 1.tif]
